# Seat belt use among rural non-drivers: the role of demographic and traffic-related variables

**DOI:** 10.5249/jivr.v16i1.1852

**Published:** 2024-01

**Authors:** Homayoun Sadeghi-Bazargani, Fatemeh Malekpour, Yousef Mohammadian, Tohid Jafari-koshki, Forouzan Rezapur-Shahkolai, Mehdi Khansari, Alireza Malekpour, Masoumeh Maleki Marzroud

**Affiliations:** ^*a*^ Road Traffic Injury Research Center, Tabriz University of Medical Sciences, Tabriz, Iran.; ^*b*^ Department of Health and Traffic, Faculty of Health, Tabriz University of Medical Sciences, Tabriz, Iran.; ^*c*^ Department of Occupational Health Engineering, Faculty of Health, Tabriz University of Medical Sciences, Tabriz, Iran.; ^*d*^ Molecular Medicine Research Center, Department of Statistics and Epidemiology, Faculty of Health, Tabriz University of Medical Sciences, Tabriz, Iran.; ^*e*^ Department of Public Health, School of Public Health, Hamadan University of Medical Sciences, Hamadan, Iran.; ^*f*^ Department of Statistics and Epidemiology, Faculty of Health, Tabriz University of Medical Sciences, Tabriz, Iran.; ^*g*^ Educational Management, Department of Educational Sciences, Faculty of Literature and Humanities, Urmia University, Urmia, Iran.; ^*h*^ Department of Education of Hashtroud, Hashtroud, Iran.

**Keywords:** Car occupants, Wearing seat belt Parents, Adherence to traffic rules, Training

## Abstract

**Background:**

The rate of seat belt use in rural societies is less than in urban societies. The present study aimed to determine the effect of demographic and traffic-related variables on seat belt use among rural non-drivers based on the theory of planned behavior (TPB).

**Methods:**

This study was conducted among 450 non-drivers in the rural areas of Hashtroud district in Iran. For collection of data, a questionnaire containing questions about demographic characteristics and general information on traffic-related behaviors of non-drivers, and questions on seat belt use based on constructs of the TPB was used.

**Results:**

The lowest seat belt use rate was for non-drivers that sit in the rear seat of a car on rural roads (22.4 % never, 14.4 % always). Also, the rate of seat belt use among parents of participants on rural roads was lower than on city roads. Adherence to traffic rules and having training about seat belt use had significant effects on the construct of TPB, including attitude, subjective norms, perceived behavioral control, behavioral intention, and behavior of seat belt use. With increasing age, subjective norms about seat belt use have improved. The attitude toward seat belt use among females was better than males.

**Conclusions:**

The result indicated that most of rural non-drivers did not adhere to traffic rules. Adherence to the traffic rules and having training on seat belt use had a significant impact on seat belt use behavior. Training seat belt use especially by parents could be effective in improving seat belt use.

## Introduction

Road traffic accident injuries (RTIs) have become a serious global public health problem that leads to the death of nearly 1.24 million people and disabling between 20 and 50 million people annually.^1^ Most RTI-related deaths occur in low-and middle-income countries.^2,3^ RTIs impose considerable expenses on governments, individuals, and their families. Iran has one of the highest rates of RTIs in the world. Based on a report by the Iranian Legal Medicine Organization (ILMO), 16778 and 317120 people died and were injured due to RTIs in 2021, respectively.^4^ According to mortality statistics in Iran, RTIs are reported as one of the main reasons for mortality in Iran.^5^ Self-report surveys have documented lower levels of rural respondents who report always using a seat belt, even after adjusting for other factors associated with restraint use, such as body mass index (BMI), education, and household income.^6,7^ Based on previous study, the rate of seat belt use among rural area residents was less than in urban residents.^8^ Previous studies have found seat belt use decreased significantly with increasing rurality.^6,8-10^

Human, environmental, and vehicle-related factors are the main causes of RTIs.^11^ The human factors such as seat belt and wearing helmet behaviors, ignoring traffic regulations and rules, and illegal speeding are the important causes of the RTIs.^12,13^ Previous studies have reported that seat belt use has an important role in reducing the number and severity of injuries.^14,15^ Forty-seven percent of passenger vehicle occupants killed in traffic crashes in 2018 were unrestrained.^16^ Seat belts prevent occupants from being thrown out of the vehicle or from hitting objects that are close to the accident area by restraining them in their seats during vehicle accidents. It is estimated that seat belt use reduces the risk of fatal injuries from vehicle crashes by approximately 45% and serious injuries by approximately 50%.^17,18^

Part of the disparity in crash deaths in urban and rural areas is likely because of differences in seat belt use among passenger-vehicle occupants.^6,7^ Half (50%) of fatally injured occupants on rural roads were unrestrained in 2015, compared with 46% of fatally injured occupants on urban roads.^19^

Studies reported that vehicle driver and passengers’ demographic factors (e.g. gender, age, educational level, and marital status),^20-24^ psychological factors,^25,26^ seating position of occupants in the vehicle (drivers, front seat or rear seat passenger),^20,24,27,28^ the strictness of legislation enforcement^20,29^ are effective factors in seat belt use. Personality is another factor in traffic safety-related behaviors of drivers.^30^

Having positive attitudes or beliefs about wearing seat belts improves the probability of wearing seat belts.^17,27^ The theory of planned behavior (TPB) is a suitable theory for the prediction of traffic safety-related behaviors. The constructs of TPB were attitude, subjective norms, behavioral intention, perceived behavioral control, and behavior. Behavioral intentions are the best predictor of behavior that is specified by attitude, subjective norms, and perceived behavioral control. These behaviors are influenced by behavioral intention and perceived behavioral control.^31^

The use of seat belts among drivers and passengers on rural roads does not seem to be at a desirable level, and the factors related to the non-use of seat belts among non-drivers of rural societies are not yet well documented. Therefore, this study sought to investigate the effect of the influence of demographic characteristics and traffic-related variables on wearing seat belts among rural non-drivers.

## Methods 

**Participants**

This cross-sectional study was conducted among non-drivers of rural areas of Hashtroud district for 2023. Hashtroud is a part of East Azarbaijan Province in Iran, it located in the northwest of Iran. The sample size was estimated using

$$ n=\frac{(z_\dfrac{a}{2})^2\Pq}{d^2} $$,

with considering 95% confidence level, and prevalence of 25% for seat belt use according to pilot study and error (d) of a maximum 4%. Eventually, the sample size was estimated as 450 non-drivers. In this study, number of non-drivers from each rural area were chosen through a simple random sampling method based on the sample size.

The inclusion criteria were being male and female as non-driver residents in the rural of Hashtroud, showing a willingness to participate in the study, and obtaining participants’ consent for participation in the study. 

**Data collection instrument**

The instrument of data collection was a researcher-made questionnaire that we designed in our previous study.^20,32^ The first section contained questions on demographic information including non-drivers' educational levels, age, and gender, and information on traffic-related behavior, such as seat belt use by parents, having experience of fine due to not using seat belts, adherence to traffic rules, opinion on mandatory seat belt use, position of sitting in the car (front or rear seat) and having training about seat belt use. The second section included questions on the non-driver’s wearing seat belt behavior based on TPB. The TPB construct were attitude, behavioral intention, subjective norms, perceived behavioral control, and behavior related to seat belt use. These second section questions were based on a 5-point Likert-type scale, and non-drivers' answers were scored from 5 to 1, representing always, most of the time, sometimes, seldom, and never using a seat belt, respectively. The higher score for a mean of TPB constructs indicates a better status. The range of scores of attitude, behavioral intention, subjective norms, perceived behavioral control, and behavior related to seat belt use were 6-30, 7-175, 8-200, 9-225, and 6-30, respectively.

**Statistical analysis**

In this study, the mean (standard deviation (SD)) and frequency (percentage) were used to present descriptive statistics of quantitative and qualitative variables, respectively. The ANOVA analysis was used to compare mean constructs of TPB among different study groups. A linear regression model was built to predict effective factors in constructs of TPB. For the analysis of data, SPSS software (version 16) was employed. P value <0.05 is considered a significant difference.

## Results

The mean (SD) age of participants was 22.68 (12.27). Table 1 indicates that 91.1% of the participants had an educational level of diploma and below. More than half of participants to some extent adhere to the traffic rules. Only 4.53 % of participants believe that wearing a seat belt is mandatory in the rear seat on rural roads. 34.4 % of non-drivers were trained in wearing seat belt by parents and friends.

**Table 1:** Frequency of participants that answered to demographic and traffic related variables (n=450).

Variable	Variable	Percent
Sex	Male	53.2
	Female	46.8
Educational level	Illiterate	0.9
	Diploma and below	91.1
	Academic	8
Adhere to the traffic rules	Strictly adhere to the traffic rules	43.4
	To some extent adheres to the traffic rules	52.3
	Not at all adhere to the traffic rules	4.2
Training in seat belt use	Not trained	21.1
	Radio and television	27.1
	Virtual spaces	15
	Friends and parents	34.3
	Others	2.5
Agree with mandatory seat belt use	Front seat inside the city	88.2
	Front seat outside the city	89.8
	Rear seat inside the city	56.6
	rear seat inside the city	69
	Front seat on rural roads	70.9
	Rear seat on rural roads	53.4
Choosing seating position	Front	36.9
	Rear	22.6
	Not important	40.5
Fine for not wearing a seat belt	Yes	29.2
	No	70.8

The lowest seat belt use rate was for non-drivers who sit in the rear seat of a car on rural roads (22.4 % never, 14.4 % always). The highest rate of seatbelt use was for non-drivers who sit in the front seat of a car outside the city (5.5 % never, 37 % always). 

Table 2 shows seat belt use behavior by parents of non-drivers on different roads and sitting positions. The lowest rate of seat belt use for both mothers and fathers was for rear seats on rural roads and outside of the city (Table 2).

**Table 2:** Percent of seat belt use by parents of non-drivers in different road and sitting positions.

Seat belt use behavior	Mother (Yes)	Mother (No)	Father (Yes)	Father (No)
Fastening seat belt as non-drivers by parent in front seat of the car inside the city	66.3	33.4	65.1	34.9
Fastening seat belt as non-drivers by parent in front seat of the car outside the city	79.5	20.5	84.4	15.6
Fastening seat belt as non-drivers by parent in front seat of the car on rural roads	59.9	39.8	61.9	38.1
Fastening seat belt as non-drivers by parent in rear seat of the car inside the city	52.2	47.8	55.5	44.5
Fastening seat belt as non-drivers by parent in rear seat of the car outside the city	62.2	37.8	71.3	28.7
Fastening seat belt as non-drivers by parent in rear seat of the car on rural roads	52.1	47.9	56.6	43.4

Results indicated that 21.10% of non-drivers were not trained for wearing seat belt on roads, 27.10%, 15%, 34.30, and 2.50 % were trained by radio and television, cyberspace, parents and friends, and others, respectively. The non-drivers who were not trained for seat belt use had a bad status for the TPB construct compared to non-drivers who were trained for seat belt use (P<0.05). ANOVA analysis demonstrated that the non-drivers that trained in seat belt use by parents and friends had favorable behavioral intention, attitude, subjective norm, perceived behavioral controls, and behavior of seat belt use compared to non-drivers that were not trained (Table 3). The higher score for the means of TPB constructs indicate a better status.

**Table 3:** Comparing the mean of TPB constructs based on training about wearing a seat belt

Construct	Training in seat belt use	Mean (SD)	P-value
Behavioral intention	Not trained	21.36 (5.61)	0.005*
	Radio and television	23.61 (5.33)	
	Cyberspace	23.73 (4.87)	
	Parents and friends	24.04 (5.29)	
	Others	23.45.36 (5.62)	
Attitude	Not trained	114.30 (35.18)	<0.001*
	Radio and television	131.36 (31.85)	
	Cyberspace	119.87 (28.15)	
	Parents and friends	131.27 (31.09)	
	Others	97.60 (15.77)	
Subjective norms	Not trained	108.50 (41.78)	0.012*
	Radio and television	122.39(40.69)	
	Cyberspace	118.02(32.13)	
	Parents and friends	131.60 (47.09)	
	Others	85.25 (36.97)	
Perceived behavioral control	Not trained	91.49 (54.60)	<0.001*
	Radio and television	120.99 (60.57)	
	Cyberspace	93.06 (54.56)	
	Parents and friends	125.45 (57.06)	
	Others	98.55 (60.62)	
Behavior	Not trained	17.28 (6.60)	0.002*
	Radio and television	20.19 (6.01)	
	Cyberspace	20.60 (5.64)	
	Parents and friends	20.14 (6.33)	
	Others	18.36 (5.29)	

*P<0.05

Results of regression analysis showed that adherence to the traffic rules and having training about seat belt use had a significant impact on attitude, subjective norms, perceived behavioral control, behavioral intention, and seat belt use behavior (P<0.05) (Table 4-8).

**Table 4:** The relationship between demographic variables and traffic-related variables with attitude to seat belt use.

Variable	B	SE	Std. beta	p-value
Constant	114.845	6.323		0.000
Sex (male)	-8.374	3.688	-0.127	0.023*
Age	-0.088	0.149	-0.035	0.556
Education level				0.756
Illiterate	Ref.	-	-	-
<diploma	-1.214	3.659	-0.019	0.740
Academic	-5.249	7.162	-0.042	0.462
Penalty	-7.775	3.810	-0.109	0.042*
Training (yes)	18.282	4.050	0.242	0.000*
Adherence to the traffic rules (yes)	11.168	3.562	0.168	0.002*
Seating position	-	-	-	0.086
Indifferent	Ref.	-	-	-
Front	-1.868	4.178	-0.026	0.655
Rear	8.051	4.366	0.106	0.066

*P<0.05

**Table 5:** The relationship between demographic variables and traffic-related variables with intention to seat belt use.

Variable	B	SE	Std. beta	p-value
Constant	20.963	1.076		0.000
Sex (male)	-0.532	0.622	-0.049	0.393
Age	-0.001	0.025	-0.003	0.954
Education level				0.540
Illiterate	Ref.	-	-	-
<diploma	-0.632	0.621	-0.059	0.310
Academic	-0.838	1.210	-0.041	0.489
Penalty	0.584	0.650	0-050	0.370
Training (yes)	2.056	0.680	0.167	0.003*
Adherence to the rules (yes)	1.987	0.603	0.182	0.001*
Seating position	-	-	-	0.269
Indifferent	Ref.	-	-	-
Front	0.394	0.707	0.033	0.578
Rear	1.206	0.744	0.096	0.106

*P<0.05

**Table 6:** The relationship between demographic variables and traffic-related variables with subjective norms about seat belt use.

Variable	B	SE	Std. beta	p-value
**Constant**	79.196	11.723		0.000
**Sex (male)**	-1.570	6.470	-0.018	0.809
**Age**	0.680	0.257	0.206	0.009*
**Education level**				0.426
Illiterate	Ref.	-	-	-
<diploma	4.528	6.434	0.052	0.482
Academic	-9.580	6.434	-0.052	0.403
**Penalty**	2.017	6.409	0.022	0.753
**Training (yes)**	20.955	7.141	0.206	0.004*
**Adherence to the rules (yes)**	16.992	6.050	0.195	0.005*
**Seating position**	-	-	-	
Indifferent	Ref.	-	-	-
Front	-8.088	6.891	-0.087	0.242
Rear	7.045	7.865	0.067	0.372

*P<0.05

**Table 7:** The relationship between demographic variables and traffic-related variables with perceived behavioral control on seat belt use behavior.

Variable	B	SE	Std. beta	p-value
**Constant**	103.492	11.909		0.000
**Sex (male)**	-4.477	6.801	-0.038	0.511
**Age**	-0.026	0.276	-0.006	0.924
**Education level**				0.034*
Illiterate	Ref.	-	-	-
<diploma	-6.616	6.846	-0.048	0.418
Academic	-33.085	13.274	-0.148	0.013*
**Penalty**	1.660	7.140	0.013	0.816
**Training (yes)**	20.387	7.600	0.148	0.008*
**Adherence to the rules (yes)**	22.101	6.595	0.186	0.001*
**Seating position**	-	-	-	0.071
Indifferent	Ref.	-	-	-
Front	-17.937	7.765	-0.138	0.022*
Rear	-6.616	8.166	-0.040	0.418

*P<0.05

**Table 8:** The relationship between demographic variables and traffic-related variables with behavior of seat belt use.

Variable	B	SE	Std. beta	p-value
**Constant**	16.992	1.239		0.000
**Sex (male)**	-0.435	0.721	-0.035	0.530
**Age**	0.017	0.029	0.035	0.553
**Education level**				0.197
Illiterate	Ref.	-	-	-
<diploma	-1.176	0.689	0.196	0.102
Academic	-1.637	1.417	-0.067	0.249
**Penalty**	-0.808	0.753	-0.058	0.284
**Training (yes)**	2.431	0.797	0.164	0.002*
**Adherence to the rules (yes)**	2.538	0.698	0.196	0.000*
**Seating position**	-	-	-	0.060*
Indifferent	Ref.	-	-	-
Front	-0.347	0.817	-0.025	0.672
Rear	1.743	0.865	0.117	0.045*

*P<0.05

The mean (SD) of attitude among participants that strictly, to some extent, and not at all adhere to traffic role were 133.66 (34.45), 120.92 (30.25), and 110.37 (30.65), respectively. The mean (SD) of subjective norms among participants that strictly, to some extent, and not at all adhere to traffic role were 131.40 (43.42), 11.85 (40.26), and 106.71 (40.07), respectively. The mean (SD) of behavioral intention among participants that strictly, to some extent, and not at all adhere to traffic role were 24.47 (5.32), 22.70 (5.26), and 21.68 (5.37), respectively. The mean (SD) of perceived behavioral control among participants that strictly, to some extent, and not at all adhere to traffic role were 124.33 (62.37), 104.11 (58.57), and 94.47 (61.90), respectively. The mean (SD) of sea belt use behavior among participants that strictly, to some extent, and not at all adhere to traffic role were 21.50 (6.18), 18.42 (5.86), and 17.47 (6.72), respectively. The variable of age had a significant effect on subjective norms about seat belt use (P=0.009). With increasing age, subjective norms about seat belt use were improved. The variable of sex demonstrated a significant impact on attitude to seat belt use (P=0.023), and the mean (SD) of attitude among male and female participants were 120.7 (33.42) and 129.65 (31.48), respectively (Table 4 - 8 ).

## Discussion

Results of the present study demonstrated that the rate of seat belt use among rural non-drivers was low, and the lowest rate of seat belt use was for non-drivers that sit in the rear seat of a car on rural roads and the highest rate of seatbelt use was for non-drivers that sit in the front seat of a car outside the city. The low rate of seat belt use among vehicle occupants is known to be one of the main reasons for traffic accident fatalities in Iran. In a study in Iran, results revealed that only 27% of drivers were ticketed for not wearing a seat belt in the past 3 years, while the rates of seat belt use were 50% and 75% on urban and rural roads, respectively, which are worryingly low compared with developed countries.^33^ Based on rural road accident statistics, during the years 2004 to 2019, the number of deaths in Iranian rural traffic-related accidents increased.^4^ Previous studies have found seat belt use decreased significantly with increasing rurality.^6,8-10^ Furthermore, the rate of car occupant fatalities increases with increasing rurality.^8,34^ Watson and Austin demonstrated that unfavorable attitudes and beliefs about seat belt use by rural drivers could be a reason for the low rate of seat belt use among rural societies. ^35^ Rezapour and Ksaiabty found that the number of lanes is another factor in seatbelt usage.^36^ Rural roads with fewer traffic and lanes are related to lower enforcement, so drivers and occupants do not wear seat belts most of the time. Cox et al. reported that drivers perceive rural roads as less risky than urban roads.^37^

Based on the results of the present study, having training about seat belt use, especially through parents and friends improved attitude, behavioral intention, subjective norms, perceived behavioral control, and behavior about seat belt use among non-drivers. According to social learning theory, people's observations and perceptions of how others are generally involved in health behaviors impact people's behavior.^38,39^ Beliefs and behaviors of children are influenced by parent’s behavior.^40^ In line with the results of the current study, Simons‑Morton et al. demonstrated that social norms could impact traffic‑related behaviors.^41^ Social support of parents prevents children’s risky behaviors and increases the adherence to traffic rules observance such as seat belts use among them.^42,43^ Dunlop and Romer reported that normative perceptions related to seat belt use behavior of friends were associated with seat belt use of boys and girls.^44^ Han demonstrated that driver seat belt use improves the rat of seat belt use by passengers.^45^ Therefore, the behavior of parents and friends has a great influence on the behavior of children. The training advantage of wearing seat belt use could be suitable in increasing awareness about seat belt use and, consequently, wearing seat belts in rural societies.

Adherence to the traffic rules had significant impact on attitude, subjective norms, perceived behavioral control, behavioral intention, and seat belt use behavior. In line with results of current study, Rezapur Shahkolai et al. found that the rate of seat belt use among students that who adhere to the traffic rules was more than students who did not adhere to the traffic rules.^20^

According to the results of the present study, 29.2 % of non-drivers were fined for not wearing a seat belt, but the experience of being fined has not had a significant impact on seat belt use behavior. Results of the present study showed that attitude toward seat belt use among non-drivers who were not fined for not wearing seat belts was better than non-drivers who had experienced being fined for not wearing seat belts. Zabihi et al. found that 76% of drivers who were fined for not wearing a seat belt claimed that it had a positive influence on their seat belt use.^33^ Increasing penalties and enforcement improved seat belt use.^33,46,47^ Lack of strict monitoring by law enforcement regarding seat belt use on rural roads could be one of the important reasons for the low rate of seat belts on rural roads.

Based on the results of the present study, gender had a significant impact on the attitude to seat belt use, and attitude to seat belt use among females were better than males. The mean of constructs of behavioral intention, subjective norms, perceived behavioral control, and behavior among females were more than males but not statistically significant. Contrary to the results of the current study, previous studies reported that risky behaviors during driving among males were significantly higher than among females.^48-51^ Shaaban and Taylor reported that male drivers have a lower rate of seat belt use compared to their female counterparts (86.9% vs. 91.8%).^52^ Several studies demonstrated that gender does not have a significant influence on seat belt use behavior.^28,33,53^ The differences between males and females could be due to biological factors, attitudes, perceptions, and behaviors across cultures.^54-56^

The variable of age had a significant impact on subjective norms about seat belt use. With increasing age, subjective norms improved about seat belt use, but the influence of age on seat belt use behavior was not significant. Zabihi et al. found that age was a predictor of seat belt use on urban and rural roads, but the impact of age on seat belt use behavior was not strong (Beta=0.24), with increasing age, due to reducing getting involved in risky behaviors and promotes a sense of accepting the responsibility consequence of action, the rate of seat belt use increased.^33^ Şimşekoğlu and Lajunen found age did not have a significant influence on seat belt use behavior.^17^ Several studies reported the effect of age on seat belt use behavior.^19,57,58^

**Strengths and limitations: **The strengths of this study were addressing the seat belt use behavior of rural non-drivers on indifferent roads that indicated a low rate of seat belt use in rural roads compared to city roads, and the necessity of enforcing the mandatory law of wearing a seat belt in rural roads. Self‑reporting is one of the limitations of the present study. Also, the inability of non-drivers to fill out questionnaires was another limitation of the study. Some of the questionnaires completed by researchers were based on oral answers from non-drivers. 

## Conclusion

Results indicated the rate of seat belt use among rural non-drivers was unfavorable. Most rural non-drivers do not adhere to traffic rules. Adherence to the traffic rules and having training on seat belt use had a significant impact on seat belt use behavior. With increasing age, subjective norms about seat belt use have improved. Attitude to seat belt use among females was better than among males. The training advantage of seat belt use could be effective in increasing seat belt use among non-drivers.
